# Risk factors for spinal subdural hematoma after minimally invasive transforaminal lumbar Interbody Fusion (MI-TLIF): a multivariate analysis

**DOI:** 10.1186/s12891-023-06902-z

**Published:** 2023-12-05

**Authors:** Jiye Lu, Wei Zhang, Guoqiang Jiang, Kefeng Luo, Kaiwen Cai, Kai Zhang, Bin Lu

**Affiliations:** Department of Spinal Surgery, The First Affiliated Hospital of Ningbo University, Work, 315000 China

**Keywords:** Postoperative spinal subdural hematoma, MI-TLIF, Logistic regression analysis, Postoperative anticoagulant therapy

## Abstract

**Background:**

Spinal subdural hematoma (SSH) is a rare cause of compression of the neutral elements of the spinal cord. However, little is known about the presentation of acute SSH after lumbar spine surgery. The reason for this may be that symptomatic SSH occurs rarely and is not given enough attention by spine surgeons. Currently, the decision to perform MRI postoperatively is more dependent on surgeon preference; therefore, no high-quality studies have been published. Our team reports our experience in the diagnosis and management of SSH after lumbar decompression and fusion surgery.

**Methods:**

We retrospectively studied 215 patients who underwent routine MRI following minimal invasive transforaminal lumbar interbody fusion (MI-TLIF) between 2020-01-01 and 2022-06-30. The patients were divided into SSH group (17 cases) and non-SSH group (198 cases) according to the occurrence of SSH. Univariate analysis and multivariate logistic regression analysis were performed to identify relevant risk factors that increase the risk of SSH postoperatively.

**Results:**

None of the patients presented with serious neurologic symptoms, such as lower extremity paralysis or cauda equina syndrome that required emergency hematoma debridement. SSH was found in 17 (7.9%) patients and non-SSH in 198 (92.1%). Factors affecting SSH were presence of hypertension, presence of diabetes and postoperative anticoagulant therapy. The significantly independent risk factor of postoperative SSH were diabetes (P = 0.008, OR: 6.988) and postoperative anticoagulant therapy (P = 0.003, OR: 8.808).

**Conclusions:**

SSH after MI-TLIF is not a rare condition, with generally no requirement of emergency evacuation. Comprehensive anti-symptomatic treatment could achieve satisfactory results. Diabetes mellitus and postoperative anticoagulant therapy are independent risk factors for SSH. Spine surgeons should hold applicability of the use of anticoagulants after lumbar surgery.

## Introduction

Spinal subdural hematoma (SSH) is a relatively uncommon condition characterized by compression of the neural elements within the spinal cord [1, 2]. It typically observed in patients with specific predisposing factors, such as vascular malformations, tumors, bleeding disorders, anticoagulation therapy, trauma, or infection [3]. Additionally, SSH can also be associated with iatrogenic injuries, including diagnostic lumbar puncture, spinal anesthesia, or lumbar spine surgery [4].

However, little is known about the presentation of acute SSH after lumbar spine surgery. The reason for this may be that symptomatic SSH occurs rarely and is not given enough attention by spine surgeons [5, 6]. Moreover, it is commonly acknowledged that certain lower extremity neurological symptoms are expected after lumbar spine surgery. Consequently, there is insufficient evidence to support routine postoperative MRI in all patients or to define the optimal time for performing MRI [3]. Currently, the decision to perform MRI postoperatively is based more on individual surgeon preference; therefore, no high-quality studies have been published.

Therefore, our team presents our experience in the diagnosis and management of SSH following lumbar decompression and fusion surgery. All cases in our study did not involve congenital vascular anomalies, coagulopathy, or incidental durotomy during surgery.

## Materials and methods

We conducted a retrospective review of the preoperative and postoperative magnetic resonance (MR) images of 215 consecutive patients who underwent minimally invasive transforaminal lumbar interbody fusion (MI-TLIF) for lumbar degenerative disorders at a single institution between July 2018 and June 2022.

The inclusion criteria were as follows: (1) age between 50 and 80 years; (2) typical clinical symptoms and signs consistent with the manifestations of degenerative lumbar disease; (3) treatment with regular conservative therapy for at least 6 months; (4) complete clinical and imaging data; (5) a minimum follow-up period of 6 months.

Patients with severe coagulation disorder, severe osteoporosis (T-score less than − 2.5), previous lumbar surgical history, spinal metastasis, rheumatic disease, and spinal infections were excluded from our analysis. Approval was obtained from our Institutional Review Board.

### Operative procedure

After general anesthesia was performed, the patients were positioned in the prone position on a radiolucent table. The pedicles of the operation level were marked using fluoroscopy. First, several small paramedian skin incisions were made and the transpedicular guide wires were inserted under C-arm fluoroscopy. Sequential dilator tubes were then inserted, and an expandable tube was placed to expose the facet joints at the responsibility level. After the facetectomy was completed, nerve root and canal decompression were performed sufficiently. Subsequently, the intervertebral disc was removed, and cartilage endplates were carefully prepared. A polyetheretherketone cage filled with autogenous bone graft (obtained from locally resected bony structures) and bone chips were implanted into the intervertebral space. Lastly, the percutaneous pedicle screws were slid over the guide wires under fluoroscopic guidance. After thorough irrigation of the surgical wounds, a drainage catheter was placed, and the wounds were closed in layers. In this research, all procedures were performed by the same senior attending physician with extensive experience, Dr. Lu Bin.

### Perioperative management

All patients were encouraged to engage in off-bed activities from the first day after the surgery with the assistance of brace. Patients without absolute contraindication received thromboprophylaxis (dalteparin sodium, 4000AxaIU, Sanofi-Aventis France) 48 h after the surgery based on the Caprini Risk Assessment Model and dynamic changes of D-dimer levels. The drainage tube was removed when the drainage volume decreased to less than 30 ml/d. X-ray and CT scans of the lumbar spine were performed after the removal of the drainage tube. An MRI examination of the lumbar spine was scheduled at day 7 after operation.

### Laboratory indicator

#### Hemoglobin

This study selected preoperative hemoglobin measurements as the evaluation parameter, with units expressed in g/L.

#### Prothrombin time, APTT, INR, PLT

This study selected preoperative prothrombin time, APTT (Activated Partial Thromboplastin Time), INR (International Normalized Ratio), and PLT (Platelet Count) as the evaluation parameters, with units as follows: Prothrombin time (seconds), APTT (seconds), INR (unitless), and PLT (10^9/L).

### Radiological assessments

MRI scans were performed using a standardized 1.5-T scanner. The evaluations of the MRI scans were conducted independently by two spine surgeons who were unaware of the patients’ clinical conditions. In cases where there was a discrepancy in their assessments, two surgeons discussed the results to reach a consensus.

We adopted the classification method proposed by Izeki et al. [3] To determine the type of SSH, axial MRI images were examined at the level with the highest occupational rate of SSH. If SSH was observed anterior to the equatorial line of the dural sac, it was classified as ventral type. Conversely, if it was located posterior to the same line, it was classified as dorsal type. When SSH was present in both areas, it was classified as ventrodorsal combined-type SSH. However, subdural lesions were not categorized as SSH-positive if they only showed linear subdural changes without a substantial volume on T2-weighted images.

### Statistical analysis

Continuous variables were presented as mean ± SD and compared using the student t-test. Nonparametric variables were compared using the Mann-Whitney U-test. Nominal variables were compared using the chi-square test. A two-tailed probability value of p < 0.05 was considered statistically significant. Multivariate logistic regression was conducted to identify independent risk factors for SSH. Risk factors with P < 0:15 in the univariate analysis were included in the model. A forward (LR) method was used, and variables with a P < 0:10 were retained in the final model, with significant variables having P < 0:05. Odds ratios (OR) and 95% confidence intervals were calculated for all variables in the model. Statistical analyses were performed using IBM SPSS (version 24, IBM Corp.).

## Results

### Incidence of SSH

A total of 48 subjects were excluded—among them 1 with severe coagulation disorder, 36 with severe osteoporosis, 5 with previous lumbar surgical history, 4 with rheumatic disease, and 2 with spinal infections. Postoperative SSH occurred in 17/215 patients (7.9%). Fortunately, none of these patients developed severe neurological deficits such as lower extremity dysfunction or bladder and bowel dysfunction that required emergency hematoma removal. 10 of the 17 patients diagnosed with SSH experienced varying degrees of lower extremity symptoms, which were effectively managed with a combination of glucocorticoids and non-steroidal anti-inflammatory drugs. Follow-up MRI scans conducted 6 months after surgery revealed spontaneous resorption of SSH in all patients.

### Risk factors

Table 1 present an overview of the demographic, hematological, and surgical-related risk factors, respectively. The analysis revealed that patients with SSH had a higher incidence of hypertension (P = 0.040), diabetes (P = 0.001), and postoperative anticoagulant therapy (P < 0.001) compared to those without SSH. However, there were no significant differences between the two groups in terms of age, sex, hematological coagulation parameters, or the number of operative levels.

**Table 1:** Comparison of patients with and without SSH

Variable	Patients with SSH (n = 17)	Patients without SSH (n = 198)	χ2/t	P value
Age	65.35 ± 9.546	65.81 ± 9.38	0.194	0.749
Sex = male	9 (52.9%)	92 (46.5%)	0.264	0.608
Hypertension	13 (76.5%)	100 (50.5%)	4.233	0.040*
Diabetes	10 (58.8%)	46 (23.2%)	10.296	0.001*
Hemoglobin	132.65 ± 17.14	138.35 ± 15.09	1.479	0.376
Prothrombin time	11.57 ± 0.33	11.41 ± 0.30	-2.107	0.598
APTT	28.49 ± 2.16	28.23 ± 1.99	-0.512	0.924
INR	1.00 ± 0.06	0.97 ± 0.51	-1.974	0.266
PLT	207.35 ± 39.60	203.85 ± 35.49	-0.387	0.746
Preoperative anticoagulant therapy	3 (17.6%)	29 (14.6%)	0.111	0.739
Postoperative anticoagulant therapy	15 (88.2%)	75 (37.9%)	16.312	<0.001*
Multilevel procedure (> 1)	8 (47.1%)	62 (31.3%)	1.768	0.184

*P < 0:05

The multivariate logistic regression analysis, using a forward (LR) method, identified that diabetes (P = 0.008, OR: 6.988) and postoperative anticoagulant therapy (P = 0.003, OR: 8.808) were independent risk factors for the development of SSH. There were no significant interactions between these risk factors (Table 2).

**Table 2:** Multivariate analysis of risk factors for SSH

Risk factor	Odds ratio	95% confidence interval		P value
Age	0	-0.065	0.064	0.991
Sex = male	0.005	-1.277	1.192	0.946
Hypertension	2.407	-2.535	0.295	0.121
Diabetes	6.988	-2.816	-0.418	0.008*
Hemoglobin	1.661	-0.069	0.014	0.198
Prothrombin time	3.103	-0.199	3.731	0.078
APTT	0.105	-0.26	0.364	0.746
INR	0.666	-7.102	17.231	0.415
PLT	0.1	-0.013	0.018	0.752
Preoperative anticoagulant therapy	0.07	-1.795	1.369	0.792
Postoperative anticoagulant therapy	8.808	-4.019	-0.822	0.003*
Multilevel procedure (> 1)	0.716	-1.712	0.68	0.397

*P < 0:05

### Appearance of the hematoma on MRI

**Fig. 1:**
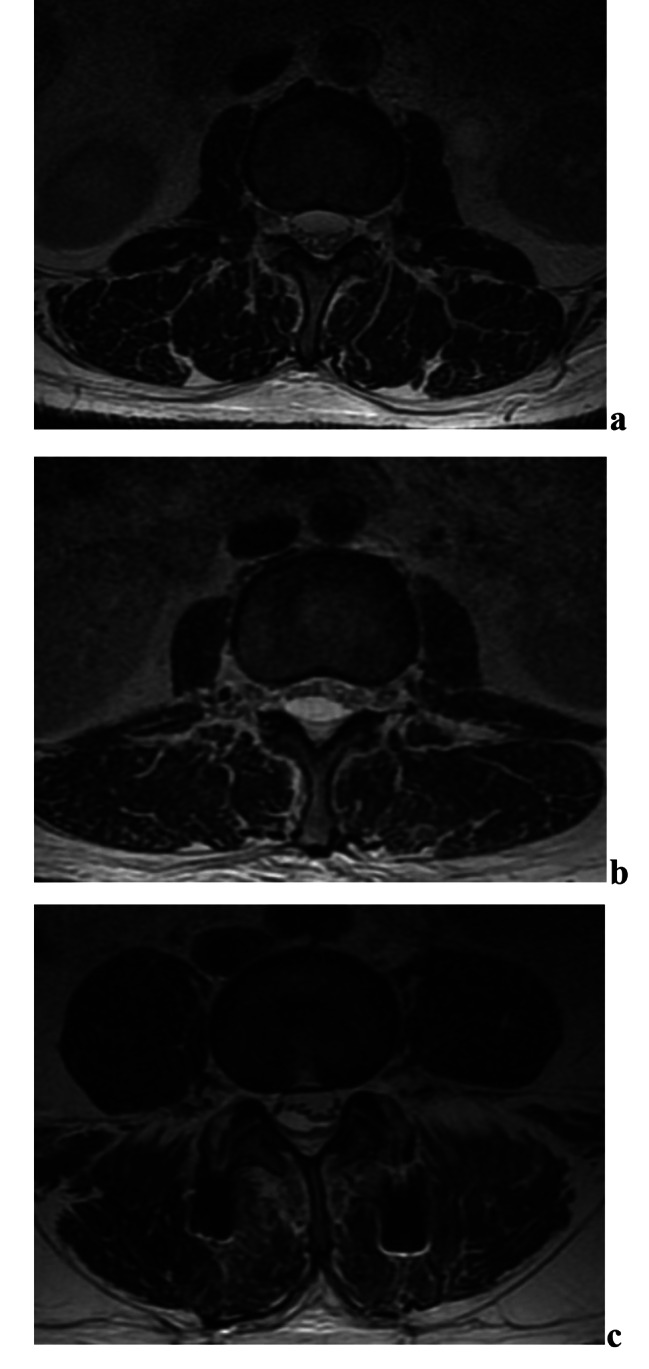
The ventrodorsal combined-type SSH. (**a**) the ventrally-type SSH; (**b**) the dorsally-type SSH; (**c**) the ventrodorsal combined-type SSH

The shape of SSH hematomas varies, including semicircular, biconvex, crescent-shaped, and also mixed shapes at different levels on axial images. The location of hematoma was predominantly located dorsally or dorsally to the cauda equina (70.6%, 12/17); however, some of the hematomas were located only ventrally (11.8%, 2/17) or combined (17.6%, 3/17) (Fig. 1).

5 patients (5/17) presented with new postoperative neurological deficits, SSH was strongly suspected as the underlying cause and other possibilities (e.g., SEH, cauda equina adhesions or recurrent disc herniation) were excluded.

### Case illustration

#### Case 1

**Fig. 2:**
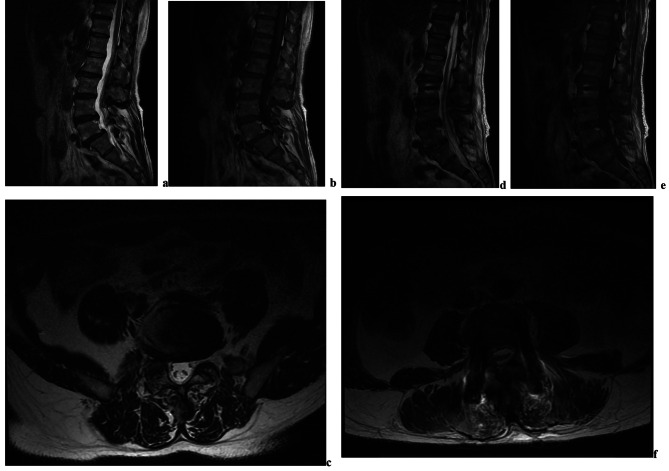
The ventrodorsal combined-type SSH. (**a**) preoperative T2-weighted sagittal magnetic resonance (MR) image; (**b**) preoperative T1-weighted sagittal MR image; (**c**) preoperative T2-weighted axial MR image; (**d**) postoperative T2-weighted sagittal MR image; (**e**) postoperative T1-weighted sagittal MR image; (**f**) postoperative T2-weighted axial MR image

A 60-year-old male with lumbar spondylolisthesis and lumbar degenerative scoliosis presented with bilateral L5 nerve root symptoms. After the surgery, the patient’s bilateral lower limb symptoms significantly improved, and no other neurological dysfunctions were detected until discharge. A scheduled MRI performed 7 days after the surgery revealed a relatively large ventrodorsal combined-type SSH extending from T12 to S1 (Fig. 2).

#### Case 2

**Fig. 3:**
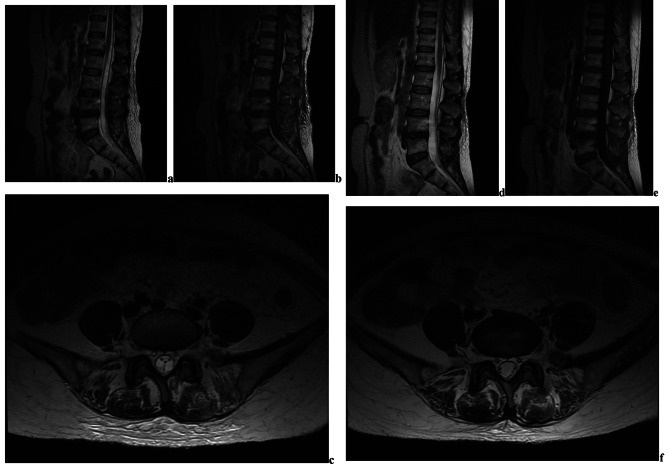
The characteristic MRI findings of SSH (the “Mercedes-Benz” sign). (**a**) postoperative T2-weighted sagittal MR image; (**b**) postoperative T1-weighted sagittal MR image; (**c**) postoperative T2-weighted axial MR image (the “Mercedes-Benz” sign); (**d**) 1 month follow-up T2-weighted sagittal MR image; (**e**) 1 month follow-up T1-weighted sagittal MR image; (**f**) 1 month follow-up T2-weighted axial MR image

A 57-year-old female with complaints of lumbar pain and left lower limb radiation pain was diagnosed with lumbar disc herniation. The lower extremity symptoms disappeared after surgery. However, 6 days after surgery, the patient gradually developed soreness and swelling in the left lower extremity, along with weakness in the left hip flexion movement. Subsequent lumbar MRI showed a large subdural hematoma with a combined ventral-dorsal SSH extending from L1 to S1, and T2-weighted images showed that the hematoma was high signal (the “Mercedes-Benz” sign). After symptomatic conservative treatment, the patient recovered completely 1 month postoperatively. A follow-up MRI performed at one month postoperatively demonstrated disappearance of subdural lesions and a restoration of normal cauda equina morphology (Fig. 3).

## Discussion

The differential diagnosis of pain and progressive neurological deficits after spinal surgery includes compression from an intradural or epidural source, infection, infarction, or other vascular factors [1]. Probably the most common of these causes is epidural hematoma, with an incidence of less than 0.3% in more than 4000 cases [7]. This rate may be even lower in cases of lumbar decompression without internal fixation. Spinal subdural hematoma (SSH) is a much less common explanation for progressive neurological deficits. It can occur spontaneous or secondary to iatrogenic injury. Spontaneous hematomas can be predisposed by trauma, infection, anticoagulation therapy, bleeding disorders, intradural tumors, or arteriovenous malformations [8].

### Our findings

None of our patients had serious neurological dysfunction and needed emergency hematoma removal. It was also explained from the side that SSH was difficult to attract enough clinical attention. Our cohort analysis found that postoperative SSH, or even asymptomatic SSH, occurred not frequently (17/ 215 patients, 7.9%). Izeki et al. [3] proposed that the incidence of postoperative SSH in minimally invasive surgery or lumbar fusion surgery with less intraoperative dural operation is much lower than that in open lumbar decompression surgery. The subjects included in this study underwent lumbar fusion under the minimally invasive channel and the incidence of postoperative SSH was 7.9%, which was lower than that reported in the literature. Through logistic multivariate analysis, we found that diabetes and postoperative anticoagulant therapy were independent risk factors for postoperative SSH.

### The occurrence and development of SHH

SHH can occur spontaneous or secondary to iatrogenic injury [9]. While iatrogenic injuries caused by lumbar puncture and epidural block have been reported in the literature, our cases did not involve clear dural tears or delayed cerebrospinal fluid leakage. Therefore, the mechanism behind postoperative SSH requires further discussion.

Currently, there are several hypotheses: the injury to the bridging vessels between dura mater and arachnoid may be the most recognized explanation for spontaneous SSH. Additionally, there exists a natural potential space between dura mater and arachnoid, called subdural space, which is connected by a delicate layer of border cells. Shear forces between the dura mater and the arachnoid disrupt the fragile cell connection, opening the subdural space, bridging vascular bleeding, and the gradual formation of a hematoma between the dura mater and the arachnoid. Although there is no apparent dural injury during the surgery, it is puzzling that the site of hematoma does not always correspond to the surgical site, and there have been reports of SSH after endoscopy. Therefore, we need to consider the mechanism more deeply. Another factor to consider is the increase in intra-abdominal pressure due to postoperative rehabilitation training or slight activities such as sneezing and coughing, may also contribute to the onset of SSH [10–12].

The spontaneous absorption of SSH can be attributed to progressive craniocaudal distribution and the decrease of hematoma pressure within the subdural space, or the dilution of hematoma by cerebrospinal fluid due to arachnoid tear [10, 13].

### Imaging findings of SHH

According to the previous studies, imaging techniques plays an essential role in the timely diagnosis and treatment of spinal subdural hematoma. Magnetic resonance imaging (MRI) and computed tomography (CT) are commonly employed for evaluating spinal hematomas [1, 4, 14, 15].

MRI is typically the preferred imaging method due to its high-resolution capabilities. It enables the detection of hemorrhage within the epidural space, evaluation of spinal cord compression, delineation of the exact levels involved, and assessment of other concomitant soft-tissue injuries [15]. Unlike epidural hematoma, subdural hematoma (SSH) is confined by the dura mater. An acute SSH appears crescent-shaped on axial MRI, while epidural hematoma tends to be more irregular. In the early stage, SSH shows high T1 signal intensity due to the presence of methemoglobin. As the SSH ages, the T1 signal intensity becomes isointense with the cerebrospinal fluid, making it difficult to detect on a T1-weighted MR image. On T2-weighted MR images, the hematoma can be detected by its mass effect on the cauda equina along with the settling of blood products within the hematoma [16]. In our patient, the hematoma appeared isodense with cerebrospinal fluid 7 days after the surgery.

Gelatin sponges are commonly used to cover defects after laminectomy and can sometimes cause confusion during MRI examinations. Blood-soaked gel sponge looks similar to the hematoma, but are characterized by not causing compression of the dural sac. In addition, axial MRI helps to differentiate between SSH and epidural hematoma. SSH extends under the dural border and is relatively regular. In contrast, an epidural hematoma appears irregular because it is not limited by the dura and can extend into the bony recess.

The “Mercedes-Benz” sign and the “cap” sign are classical imaging findings in spinal subdural hematoma (Fig. 3) [17]. The former is a tri-radiate hypointense image that resembles the Mercedes-Benz symbol on T2-weighted MRI, caused by exiting nerve roots on the sides and the clumped rootlets in the subarachnoid space posteriorly. On the other hand, the Cap sign appears as a cap of epidural fat of slightly different hyperintensity outside the black line of the dura. These imaging findings are considered classical indicators of SSH and are valuable in its identification and diagnosis.

### Risk factors of SHH

Similar to findings reported internationally, our study also confirmed that anticoagulant therapy were independent risk factors for SSH. However, the role of diabetes in SSH remains unclear. Izeki et al. noted a slightly higher incidence of diabetes (23.3%) in patients with SSH compared to those without (12.9%), but this difference wasn’t statistically significant (P = 0.102) [3]. It’s worth mentioning that diabetes could raise the risk of hypertension, which might indirectly increase the risk of SSH. Nonetheless, more research is needed to clarify the direct relationship between diabetes and SSH.

Surgery-related trauma was another contributing factor. Gehri et al. [4] attributed the occurrence of SSH to a minor dural tear during surgery, while Reinsel et al. [8] hypothesized that accidental trauma during preoperative myelography or discectomy led to SSH. In our case, the patient did not have any of these predisposing factors. However, it is important to note that the dura undergoes blunt stripping during the surgery, which can cause no small trauma to the delicate dura. Moreover, less visualization under minimally invasive access and more intraoperative traction on the dura mater also elevate the risk of injury [4].

### Clinical intervention of SHH

The early and accurate diagnosis of spinal subdural hematoma is essential, as a delay in treatment can have grave consequences, including permanent neurologic deficit or even death [2] Treatments include laminectomy with clot evacuation [18] for cases involving significant neurological deficits, or conservative management if the hematoma is small or symptoms are improving.

Drawing from Merter’s study [19], it becomes evident that early intervention can be decisive; patients with a hematoma area larger than the dural sac area who did not undergo hematoma evacuation generally recorded lower JOA scores in all subsequent assessments. Given this, it is recommended to consider emergency dural incision as an appropriate treatment strategy for acute or subacute SSH to enhance postoperative outcomes [4, 7].

Our study aims to increase awareness of the potential occurrence of SSH following lumbar decompression surgery. Additionally, spine surgeons should recognize that postoperative incapacitating lower extremity pain, which responds poorly to conventional analgesic doses and is not proportional to the expected outcome of surgical trauma, it may be a sign of acute SSH [3] Immediate symptomatic treatment with hormones and nonsteroidal anti-inflammatory drugs (NSAIDs) is recommended to protect neurological function, and emergency hematoma removal should be performed if necessary [20, 21].

### Spinal postoperative anticoagulant regimen

The current clinical attitude towards anticoagulation after spinal surgery lacks global consistency. There is a lack of consensus on various aspects of thromboprophylaxis in spine surgery [22] Surgeons in the global survey showed that their decisions were based heavily on expert opinion and fellowship training, rather than formal guidelines or literature. A global survey involving 316 spine surgeons from 64 countries [23] highlighted that decisions concerning anticoagulation therapy were based heavily on expert opinion and fellowship training, rather than formal guidelines or literature. The North American Spine Society (NASS) attempted to create clinical guidelines on antithrombotic therapies in spine surgery, which were comprehensive but have yet to be widely adopted [24]. According to the NASS guideline, most elective spine surgeries performed through a posterior approach are associated with a very low risk of venous thromboembolic events (VTEs), and chemoprophylaxis may not be warranted due to the associated risks of serious wound and bleeding complications. The American College of Chest Physicians recommends initiating chemoprophylaxis between 1 day before and 3 days after surgery [25]. Mechanical compression may also be used just before or at the beginning of surgery and continued until the patient is fully ambulatory. In line with these recommendations, our team finds it reasonable to initiate prophylaxis with LMWH 48 h after surgery. Additionally, the use of physical pressure therapy is also recommended alongside pharmacologic prophylaxis.

In our study, we identified a significant correlation between postoperative spinal subdural hematoma and anticoagulant therapy, although no serious complications were observed. This finding is supported by previous literature, where Izeki et al. [3] reported preoperative anticoagulant therapy as a risk factor for spinal subdural hematoma following lumbar decompression surgery. Another case report by Raymaekers et al. [9] described an unusual case of acute intradural hematoma after lumbar spinal surgery without evidence of dural injury. The treatment approach for spinal subdural hematoma depends on the severity of neurologic deficits, typically requiring surgical intervention [26].

Special attention should be given to patients with a history of hypertension or those receiving preoperative anticoagulant therapy as they are at higher risk. It is crucial for spine surgeons to carefully consider these risk factors and take into account each patient’s unique clinical characteristics when determining the most suitable anticoagulation therapy.

### Limitations

We must acknowledge the limitations of our study. Importantly, this is a retrospective study on patients from a single institution. The incidence of SSH across different diseases was not demonstrated in this research. Variations in intraoperative decompression techniques and the duration of the surgery can both influence the occurrence of SSH, which requires further study. Also, the relationship between the size and extent of the hematoma and the patient’s clinical symptoms remains unclear, necessitating specialized assessment. The indications and duration for the use of anticoagulant medications after spinal surgery have not yet reached a consensus. Therefore, a high-quality prospective study is much needed to clarify the aforementioned issues to better guide the clinical practice of spine surgeons.

## Conclusions

SSH after MI-TLIF is not a rare condition, with generally no requirement of emergency evacuation. Comprehensive anti-symptomatic treatment could achieve satisfactory results. Diabetes mellitus and postoperative anticoagulant therapy are independent risk factors for SSH.

## Data Availability

The data and materials used and analyzed during the current study are available from the corresponding author on reasonable request.
